# The Usefulness of the Alpha-Retroflex Position in Biliary Cannulation on Single-Balloon Enteroscopy-Assisted Endoscopic Retrograde Cholangiopancreatography in Patients with Roux-en-Y Gastrectomy: A Retrospective Study

**DOI:** 10.1155/2023/6678991

**Published:** 2023-08-04

**Authors:** Hiroo Imazu, Rota Osawa, Koji Yamada, Toshimi Takahashi, Muneo Kawamura, Shuzo Nomura, Suguru Hamana, Noriyuki Kuniyoshi, Mariko Fujisawa, Kei Saito, Hirofumi Kogure

**Affiliations:** ^1^Division of Gastroenterology and Hepatology, Department of Medicine, Nihon University School of Medicine, Tokyo, Japan; ^2^Department of Surgery and Endoscopy, Kawamura Hospital, Shizuoka, Japan

## Abstract

**Introduction:**

Balloon enteroscopy-assisted endoscopic retrograde cholangiopancreatography (BE-ERCP) is a useful therapeutic procedure that provides promising results in patients with surgically altered anatomy. However, biliary cannulation in BE-ERCP remains challenging. Therefore, in patients with Roux-en-Y gastrectomy, this study aimed to evaluate a BE-ERCP cannulation strategy that includes the newly developed alpha-retroflex scope position.

**Methods:**

This was a retrospective review of 52 patients with Roux-en-Y gastrectomy who underwent BE-ERCP at two centers between April 2017 and December 2022. In these patients, three types of scope position had been used for biliary cannulation: straight (S-position), J-retroflex (J-position), and alpha-retroflex (A-position). First, the S-position was used for biliary cannulation. Then, if biliary cannulation was difficult with this position, the J-position was used, followed by the A-position, if necessary.

**Results:**

The biliary cannulation success rate was 96.6% (50/52). The S-, J-, and A-positions achieved successful biliary cannulation in 24 (48%), 14 (28%), and 12 patients (24%), respectively. No adverse events, including post-ERCP pancreatitis and perforation, occurred.

**Conclusion:**

This was the first study of a cannulation strategy that included the A-position in addition to the S- and J-positions. The study showed that the A-position is feasible and safe in BE-ERCP in patients with Roux-en-Y gastrectomy.

## 1. Introduction

Recent advances in balloon enteroscopy (BE) enable access to the major duodenal papilla in patients with surgically altered anatomy (SAA), such as those with Roux-en-Y reconstruction after gastrectomy, and studies have reported on the usefulness of BE-assisted endoscopic retrograde cholangiopancreatography-related procedures (BE-ERCP) in patients with SAA [1–3]. BE-ERCP is becoming a safe and promising therapeutic procedure for pancreaticobiliary pathology with SAA. However, the therapeutic success rate of BE-ERCP is relatively low [1–3].

One contributing factor to the unsatisfactory therapeutic success rate of BE-ERCP is the difficulty of biliary cannulation [4, 5]. Successful biliary cannulation requires an appropriate en face view of the papilla, in which the axis of the cannulation catheter is aligned with the axis of the bile duct [6]. In the case of BE-ERCP in patients with SAA, the characteristics of a forward-viewing enteroscope without an elevator and the inverted endoscopic view of the papilla make it difficult to obtain an en face view of the papilla, which complicates biliary cannulation and thus results in a low success rate of biliary cannulation [5]. Moreover, because of the difficulties of biliary cannulation, rescue cannulation methods, such as pancreatic guidewire placement and the precut method, are often needed to achieve successful biliary cannulation in BE-ERCP [7, 8].

Generally, whether or not an en face view of papilla can be obtained in BE-ERCP is determined by the scope position. Several studies reported on the usefulness of the endoscopic retroflex position, in which the distal portion of the enteroscope under fluoroscopy becomes shaped like the letter J (referred to as the J-retroflex position or J-position), at the inferior duodenal angle as one method to obtain an en face view of the papilla in BE-ERCP [4, 5, 9, 10]. These studies showed that the J-position significantly facilitates successful biliary cannulation and enables complete stone extraction [4, 10] because it allows the axis of the cannulation catheter to be aligned with the bile duct. However, the J-position is not feasible in all patients [4, 9]. In addition to the J-position, the straight scope position (S-position), in which the distal portion of the enteroscope straightens, is often applied for biliary cannulation [4]. The straight position is easy to obtain and is the first scope position when the tip of the scope reaches the papilla; however, the S-position often shows a tangential view of the papilla, which may make biliary cannulation difficult.

To overcome the difficulties of biliary cannulation in BE-ERCP, we developed the alpha-retroflex scope position (A-position) as a new endoscopic position to obtain an en face view of the papilla. In the A-position, the distal portion of the endoscope becomes shaped like the letter alpha.

To date, no studies have reported on cannulation strategy from the perspective of scope position. Therefore, this retrospective study aimed to evaluate the outcome of cannulation in BE-ERCP when the A-position was used in addition to the S- and J-positions.

## 2. Methods

### 2.1. Study Design and Patients

The study was a retrospective review of medical records of patients with Roux-en-Y gastrectomy who underwent biliary BE-ERCP at two sites (Nihon University Hospital, Tokyo, Japan, and Kawamura Hospital, Shizuoka, Japan) between April 2017 and December 2022. All procedures were performed by an endoscopist (H.I.) with more than 25 years of experience who had been involved in more than 500 ERCPs annually as an operator or supervisor. The study included patients with native papilla and Roux-en-Y gastrectomy who underwent biliary BE-ERCP because of cholangiography, biliary drainage, and stone extraction with endoscopic sphincterotomy or papillary balloon dilation. Patients with previous successful ERCP or pancreatic ERCP were excluded.

The study was approved by our institutional review board and registered with the University Medical Information Network (registration number: 000050913). The need for informed consent was waived given the retrospective and observational nature of the study.

### 2.2. Procedure with BE-ERCP and Cannulation Strategy

**Figure 1:**
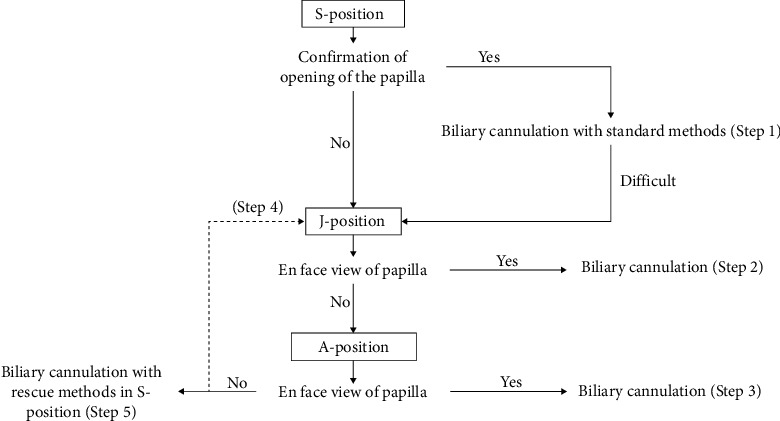
Cannulation strategy at our institution. S-position, straight scope position; J-position, J-retroflex scope position; A-position, alpha-retroflex position.

In all patients, a short-type single-balloon enteroscope (SIF-H290S, Olympus Medical Systems, Tokyo, Japan) was used for BE-ERCP. After peroral insertion, the enteroscope was advanced towards the papilla beyond the Roux-en-Y anastomosis, and after it reached the papilla, three types of scope position, that is., the S-, J-, and A-positions, were sequentially used to obtain an en face view of the papilla. Since the working channel of the single-balloon enteroscope is located at the 7 o'clock position, manipulating the enteroscope to obtain an en face view of the papilla means manipulating the enteroscope so that the axis of the cannulation catheter at the 7 o'clock position of the endoscopic image is aligned with the axis of the bile duct. The strategy of biliary cannulation with the S-, J-, and A-positions used at our institutions is shown in Figure 1. The standard biliary cannulation method used in the patients comprised a wire-guided or contrast-guided cannulation technique with a standard catheter (MTW catheter, MTW Endoskopie, Wesel, Germany) and guide wire (VisiGlide2, Olympus Medical Systems).

**Figure 2:**
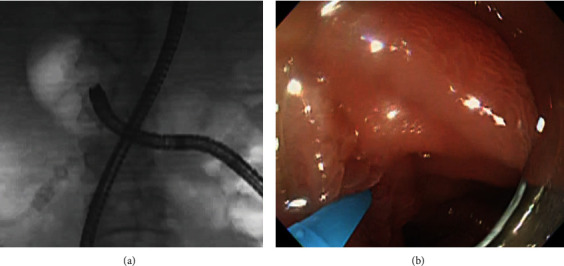
(a) Fluoroscopic and (b) endoscopic findings in the straight scope position (S-position). The S-position is likely to provide a tangential view of the papilla.

The S-position, in which the distal portion of the scope has a straight shape in fluoroscopy, was used as the first position for biliary cannulation (Step 1) because it does not require any specific manipulation of the scope when it first reaches the papilla. However, the S-position is likely to show a tangential view of the papilla because in patients with SAA, the axis of the scope tends to be parallel to the duodenal wall in the forward view (Figure 2) [1, 3–5, 7, 8, 10]. If the opening of the papilla was not visualized, or biliary cannulation with the standard cannulation method was found to be difficult in the S-position, the endoscopist attempted to place the scope in the J-position.

**Figure 3:**
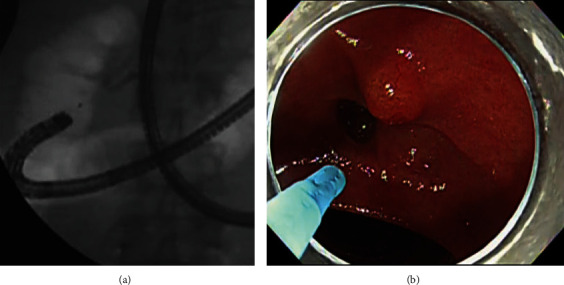
(a) Fluoroscopic and (b) endoscopic findings in the J-retroflex scope position (J-position). Here, the J-position provided an en face view of the papilla.

To create the J-position, the enteroscope was carefully advanced with an upward angulation, whereas the flexible part of the enteroscope was pressed onto the duodenal wall opposite the inferior duodenal angle. Generally, the J-position easily results in an en face view of the papilla (Figure 3) [4, 5, 9, 10]; after obtain an en face view, biliary cannulation was attempted by the standard cannulation method, followed by a rescue cannulation method (Step 2). As the rescue cannulation method, either the pancreatic guide wire placement method [11] or the rotatable sphincterotome (TRUEtomeTM RX, Boston Scientific Japan, Tokyo, Japan) method [12] was performed.

**Figure 4:**
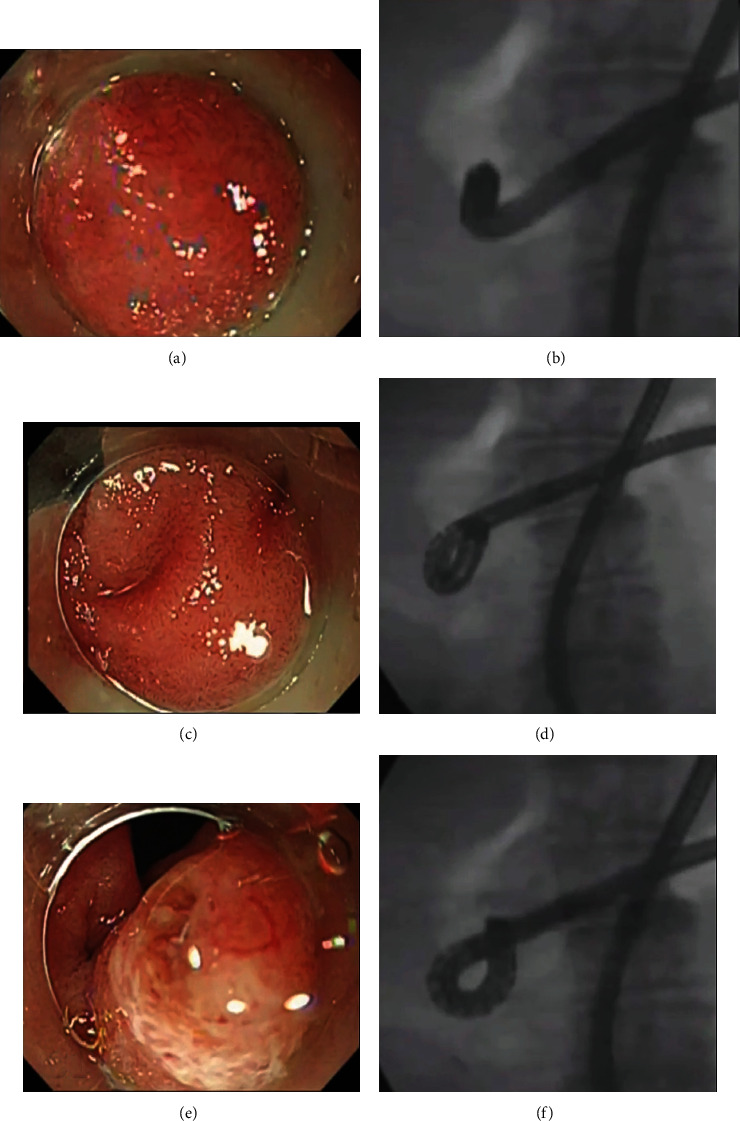
Creation of the alpha-retroflex scope position. The scope is manipulated to obtain upward angulation (a and b) and rotated clockwise (c and d), whereas the flexible part of the scope is pressed onto the duodenal wall opposite side of the inferior duodenal angle. By combining these steps with rightward angulation, the distal portion of the scope becomes shaped like the letter alpha (e and f). Here, the alpha-retroflex position provided an en face view of the papilla, although the endoscope tip was probably close to the papilla.

If an en face view of the papilla was not feasible in the J-position, as the next step the endoscopist attempted to place the scope in the A-position. To create the A-position, the scope was carefully advanced with an upward angulation, rotated clockwise, and a rightward angulation, while the flexible part of the scope was pressed onto the duodenal wall opposite the inferior duodenal angle (Figure 4). After obtaining an en face view of the papilla in the A-position, biliary cannulation was attempted by the standard cannulation method, followed by a rescue cannulation method (Step 3).

**Figure 5:**
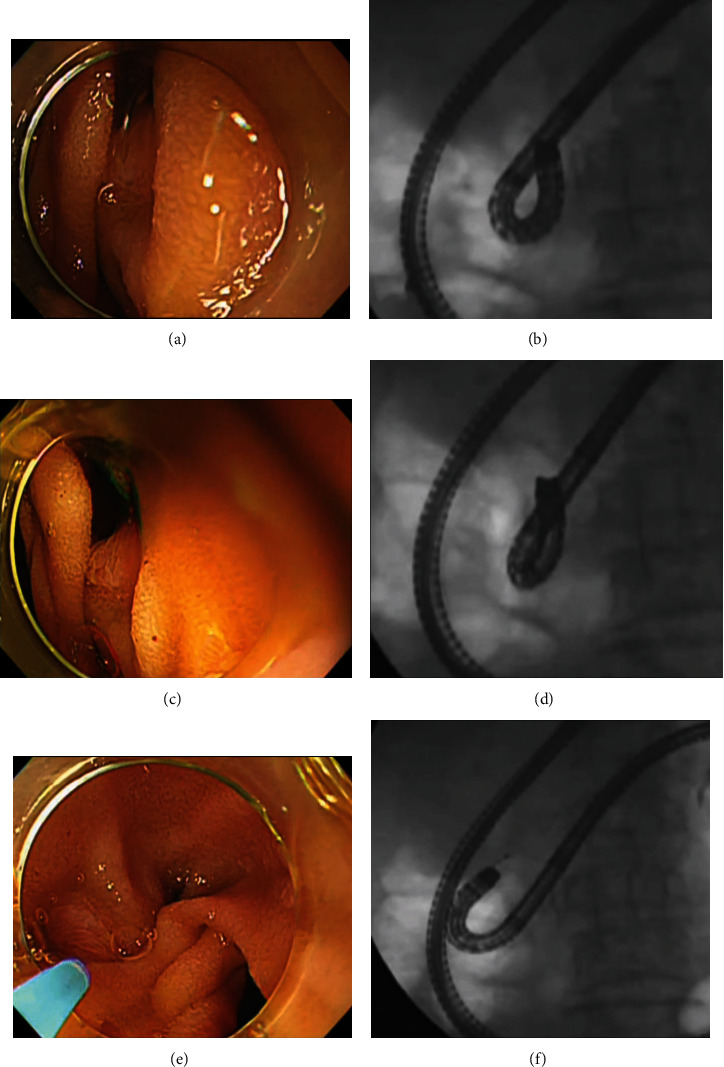
Creation of the J-retroflex position from the alpha-retroflex position. (a and b) Insufficient en face view of the papilla in alpha-retroflex position (A-position). (c and d) Fluoroscopic and endoscopic view during counterclockwise rotation and leftward angulation of the balloon enteroscope, with the flexible part of scope used as the fulcrum of rotation. (e and f) Fluoroscopic and endoscopic view in the J-retroflex position created from the A-position.

If the en face view of the papilla in the A-position was insufficient to achieve biliary cannulation, the endoscopist attempted again to create the J-position by counterclockwise rotation and leftward angulation of the scope, with the flexible part of the scope as the fulcrum of rotation (Step 4; Figure 5). If the en face view of the papilla could not be obtained with either the J- or the A-position, the S-position was created again, and biliary cannulation was attempted by a rescue cannulation method (Step 5). After deep biliary cannulation was achieved, cholangiography, endoscopic sphincterotomy, endoscopic papillary balloon dilation, endoscopic lithotomy, or endoscopic biliary drainage (EBD) was performed as required. In cases of endoscopic lithotomy, endoscopic naso-biliary drainage (ENBD) was performed after stone extraction to prevent post-ERCP cholangitis, and then, the ENBD tube was removed within 24 hours after the placement. In addition, prophylactic pancreatic stenting was performed to prevent post-ERCP pancreatitis in cases of pancreatic guide wire placement method.

### 2.3. Outcome Measurement and Definitions

Clinical and BE-ERCP-related data, such as serum amylase level before and after the procedure, clinical symptoms, failure or success of biliary cannulation, and cannulation method, were collected from the patients' medical and endoscopic records and analyzed. Wire-guided and contrast-guided cannulation with a standard catheter and guide wire were defined as the standard cannulation technique, and pancreatic guide wire and rotatable sphincterotome method were defined as rescue cannulation techniques. The diagnosis of post-ERCP pancreatitis was made according to the criteria of Cotton et al. [13].

## 3. Results

A total of 52 patients met the inclusion criteria and were included in the study. Patient characteristics are summarized in Table 1. The success rate of reaching the papilla was 100% (52/52), and the success rate of biliary cannulation was 96.2% (50/52). Biliary cannulation failed in two patients in whom the papilla was located deep within the diverticulum and the opening of the papilla could not be visualized with any scope position. After successful cannulation, the procedure success rate was 100% (50/50). In the two patients in whom biliary cannulation failed, endosonography-guided hepaticogastrostomy and hepaticojejunostomy were successfully performed. The outcome of cannulation with the three types of position according to the steps shown in Figure 1 was as follows: successful biliary cannulation was achieved in 18 patients at Step 1, 12 at Step 2, 12 at Step 3, 2 at Step 4, and 6 at Step 5. The S-, J-, and A-positions were used to achieve successful biliary cannulation in 24 (48%; Steps 1 and 5), 14 (28%; Steps 2 and 4), and 12 patients (24%; Step 3), respectively. The A-position was used directly or indirectly (i.e., as an intermediate step to using the J-position) to achieve successful biliary cannulation in 14 patients (28%; Steps 3 and 4); however, when therapeutic procedures were performed, switching to the S- or J-positions was sometimes needed after biliary cannulation in the A-position because the scope tip was too close to the papilla to allow therapeutic procedures to be performed in the A-position. Furthermore, such scope position switching was also necessary when rigid devices, such as basket catheters, could not be advanced through the working channel in the torqued scope tip. Details of the procedures are shown in Table 2.

No procedure-related adverse events, including post-ERCP pancreatitis, post-ERCP cholangitis, bleeding, and duodenal perforation, occurred in any of the 52 patients.

## 4. Discussion

BE-ERCP for patients with SAA and pancreaticobiliary pathology was recently developed and has been broadly studied [1–3]. Meanwhile, two kinds of balloon enteroscope, the single- and double-balloon enteroscope, are available for BE-ERCP, and comparison studies have shown that these scopes are equally successful in reaching the papilla and performing therapeutic BE-ERCP [14, 15]. The latest systemic review and meta-analysis of 53 studies on BE-ERCP for patients with SAA showed a successful duct cannulation rate of 74.7%, a therapeutic success rate of 69.1%, and an adverse event rate of 5.7% [2]. Another systemic review of 21 studies on single-BE-assisted ERCP reported a biliary cannulation rate of 86.6%, a therapeutic success rate of 75.8%, and an adverse event rate of 6.6% [3]. Thus, the development of the balloon enteroscope has led to a dramatic improvement in the success of ERCP-related procedures for patients with SAA. However, studies have also highlighted the limitations of BE-ERCP, such as the low success rate of biliary cannulation and therapeutic procedures [1–5]. These less-than-satisfactory results are related to the difficulty in obtaining an en face view of the papilla (which is needed to allow the axis of the catheter to be aligned with the bile duct) as a result of SAA and the properties of a forward-viewing enteroscope without an elevator [5, 8, 10].

**Figure 6:**
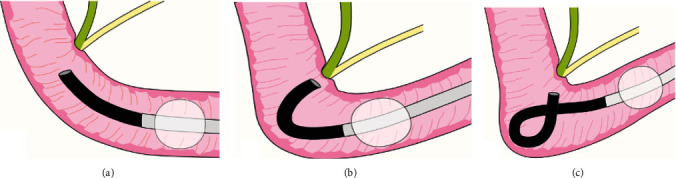
Schema of balloon enteroscope position. (a) Straight position (S-position). (b) J-retroflex position (J-position). (c) Alpha-retroflex position (A-position). The S-position provides a tangential view of the papilla, and the J-position provides an en face view. The A-position also provides an en face view of the papilla, although the scope tip is likely to be close to the papilla. S-position, straight scope position; J-position, J-retroflex position; A-position, alpha-retroflex position.

Thus, the key to successful biliary cannulation in BE-ERCP is obtaining an en face view of the papilla during enteroscopy. The endoscopic view of the papilla in BE-ERCP is often determined by the scope position. Traditionally, the S- or J-positions have been used for biliary cannulation. Although it is technically very easy to place the scope in the S-position, this position often provides a tangential view of the papilla, which can make biliary cannulation difficult (Figure 6(a)). Several studies showed that the J-position could provide an en face view of the papilla and facilitate biliary cannulation [4, 10] (Figure 6(b)), but we often encountered cases in which the scope tip advanced beyond the papilla while attempting to create the J-position, resulting in an S-position. Thus, the J-position is not always feasible in all patients. Because the S- and J-positions alone do not always provide an en face view of the papilla, we developed the A-position as an alternative. By performing upward angulation, clockwise rotation, and rightward angulation of the scope, the distal portion of the scope becomes shaped like an inverted J, that is, an alpha shape, and this so-called A-position allows an endoscopic view upwards to the papilla, with the scope tip close to the papilla (Figure 6(c)). Moreover, even in cases where the first attempt to create the J-position failed, we were sometimes above to create it from the A-position by counterclockwise rotation of the scope combined with leftward angulation, thereby using the flexible part of the scope as the fulcrum of rotation. Based on this concept, we developed the A-position and cannulation strategy for BE-ERCP in patients with Roux-en-Y gastrectomy, as shown in Figure 1. This was the first study of the cannulation strategy with the A-position in BE-ERCP.

The rate of successful biliary cannulation in our cannulation strategy, which used the A-position in addition to the S- and J-positions, was 96.6% (50/52). Except for two patients in whom the papilla was not endoscopically visualized because they had a large diverticulum, the rates of successful biliary cannulation and successful procedures were 100% (50/50) and 100% (50/50), respectively, which are comparable with or better than the rates found in the latest meta-analysis [2] and systemic review [3]. The A-position was used for successful biliary cannulation in 24% of patients (Step 3), although biliary cannulation was successfully achieved with the S- or J-positions in almost all patients. In addition, because a J-position created from the A-position was used for successful biliary cannulation in 2 patients, the A-position was directly or indirectly useful in the achievement of biliary cannulation in 28% of patients (Steps 3 and 4). No adverse events, including post-ERCP pancreatitis and perforation, occurred in this study, so our results show that the A-position is feasible and safe and that a cannulation strategy that includes the A-position can improve the rate of successful biliary cannulation for BE-ERCP in patients with Roux-en-Y gastrectomy. One should note that the creation of the A-position was often accompanied by complicated scope manipulation, so this procedure should be performed carefully to avoid causing perforation. In addition, switching to the S- or J-positions was sometimes needed after biliary cannulation in the A-position, probably because in the A-position, the scope tip was too close to the papilla to allow therapeutic procedures to be performed.

Meanwhile, single- and double-balloon enteroscopes are available for BE-ERCP, and both have been reported to have the same success rates in reaching the papilla and performing therapeutic BE-ERCP [14, 15]. In the present study, a single-balloon enteroscope was used for biliary cannulation. The main difference between a single- and double-balloon enteroscope that could affect the results of biliary cannulation is that only the single-balloon enteroscope allows passive bending and high-force transmission. In our experience, when using a single-balloon enteroscope, the tip of the scope can easily be advanced to the deep part of the duodenum beyond the papilla when upwards angulation is used to create the J-position at the inferior duodenal angle. Hence, the A-position might be needed more often in BE-ERCP when using a single-balloon enteroscope than when using a double-balloon enteroscope.

This study has several limitations. First, it was a retrospective study with small sample size and without a comparative cohort. In addition, all procedures were performed by a well-experienced endoscopist. The indication for BE-ERCP was choledocholithiasis in almost all of the patients, and only two patients had malignant disease. If the study had included patients in whom BE-ERCP had been performed by physicians with various levels of experience and patients with malignant disease, the outcomes might have been different because the creation of the A-position often requires precise endoscopic manipulation. However, the high incidence of choledocholithiasis in the indication for BE-ERCP in this study might be reasonable, because the frequency of biliary tract stones is higher in patients who have undergone gastrectomy than in the general population [16]. Multicenter, prospective, randomized comparison studies on cannulation with and without the A-position are needed to clarify the usefulness of the A-position in BE-ERCP.

## 5. Conclusion

In conclusion, this first study of a cannulation strategy that used the A-position in addition to the S- and J-positions showed that the A-position helps to overcome the difficulties of biliary cannulation in BE-ERCP and enables an en face view of the papilla to be obtained, thus facilitating biliary cannulation. The study found that the A-position is feasible and safe for BE-ERCP in patients with Roux-en-Y gastrectomy.

## Figures and Tables

**Table 1:** Characteristics of patients with SAA undergoing biliary cannulation for balloon enteroscopy-assisted endoscopic retrograde cholangiopancreatography.

Patients with native papilla and Roux en-Y gastrectomy, *n*	52
Age, mean ± SD (years)	78.1 ± 7.8
Sex, M/F	39/13
Indications	
Choledocholithiasis	50
Klatskin's tumor	2

**Table 2:** Outcomes of a biliary cannulation strategy with three types of scope position.

Success rate of reaching the papilla, (*n*/*N*)%	100 (52/52)
Successful biliary cannulation rate, (*n*/*N*)%	96.2 (50/52)
Procedure success rate, (*n*/*N*)%	100 (50/50)
Scope position and cannulation method, *n*(%)	
S-position	24 (48%)
Standard method (Step 1)	18
Rescue method (Step 5)	6
J-position	14 (28%)
Standard/rescue method (Step 2)	11/1
J-position from A-position with standard method (Step 4)	2
A-position	12 (24%)
Standard/rescue method (Step 3)	11/1
Endoscopic procedures	
EST + stone extraction	1
EPBD + stone extraction	14
EBD	32
Cholangiography	3
Adverse event	
Post-ERCP pancreatitis	0
Others	0

A-position, alpha-retroflex position; EBD, endoscopic biliary drainage; EPBD, endoscopic papillary balloon dilation; ERCP, endoscopic retrograde cholangiopancreatography; EST, endoscopic sphincterotomy; J-position, J-retroflex position; S-position, straight position.

## Data Availability

Data supporting this research article are available from the corresponding author on reasonable request.
